# Fatty liver classification via risk controlled neural networks trained on grouped ultrasound image data

**DOI:** 10.1038/s41598-024-57386-3

**Published:** 2024-03-28

**Authors:** Tso-Jung Yen, Chih-Ting Yang, Yi-Ju Lee, Chun-houh Chen, Hsin-Chou Yang

**Affiliations:** Institute of Statistical Science, Academia Sinica, Taipei, Taiwan

**Keywords:** Health care, Medical imaging, Ultrasonography, Mathematics and computing, Scientific data, Statistics

## Abstract

Ultrasound imaging is a widely used technique for fatty liver diagnosis as it is practically affordable and can be quickly deployed by using suitable devices. When it is applied to a patient, multiple images of the targeted tissues are produced. We propose a machine learning model for fatty liver diagnosis from multiple ultrasound images. The machine learning model extracts features of the ultrasound images by using a pre-trained image encoder. It further produces a summary embedding on these features by using a graph neural network. The summary embedding is used as input for a classifier on fatty liver diagnosis. We train the machine learning model on a ultrasound image dataset collected by Taiwan Biobank. We also carry out risk control on the machine learning model using conformal prediction. Under the risk control procedure, the classifier can improve the results with high probabilistic guarantees.

## Introduction

Fatty liver is a condition in that fat are accumulated excessively in the liver cells. It is a disease that can lead to chronic inflammation, irreversible cirrhosis, liver failure and liver cancer. It is commonly-seen in Asia. For example, in Taiwan around 11.5% of adult population has fatty liver. A recent study^1^ pointed out that in Asia around 0.53% of fatty liver patients die directly due to the disease every year. Given its popularity and severity, it is important to run early diagnosis and intervention on fatty liver disease to prevent its progression and improve its prognosis.

Several methods are available for fatty liver diagnosis. These methods include liver biopsy, blood tests, and imaging scans such as those using ultrasound or CT technologies. While liver biopsy is considered as the gold standard for fatty liver diagnosis, it is an invasive procedure that may cause some risks. To avoid such risks, non-invasive methods such as ultrasound imaging is a popular choice. It uses a probe to transmit ultrasound pulses to the targeted organ. Once the pulses are reflected from the targeted organ, an image of the organ will be created.

**Figure 1:**
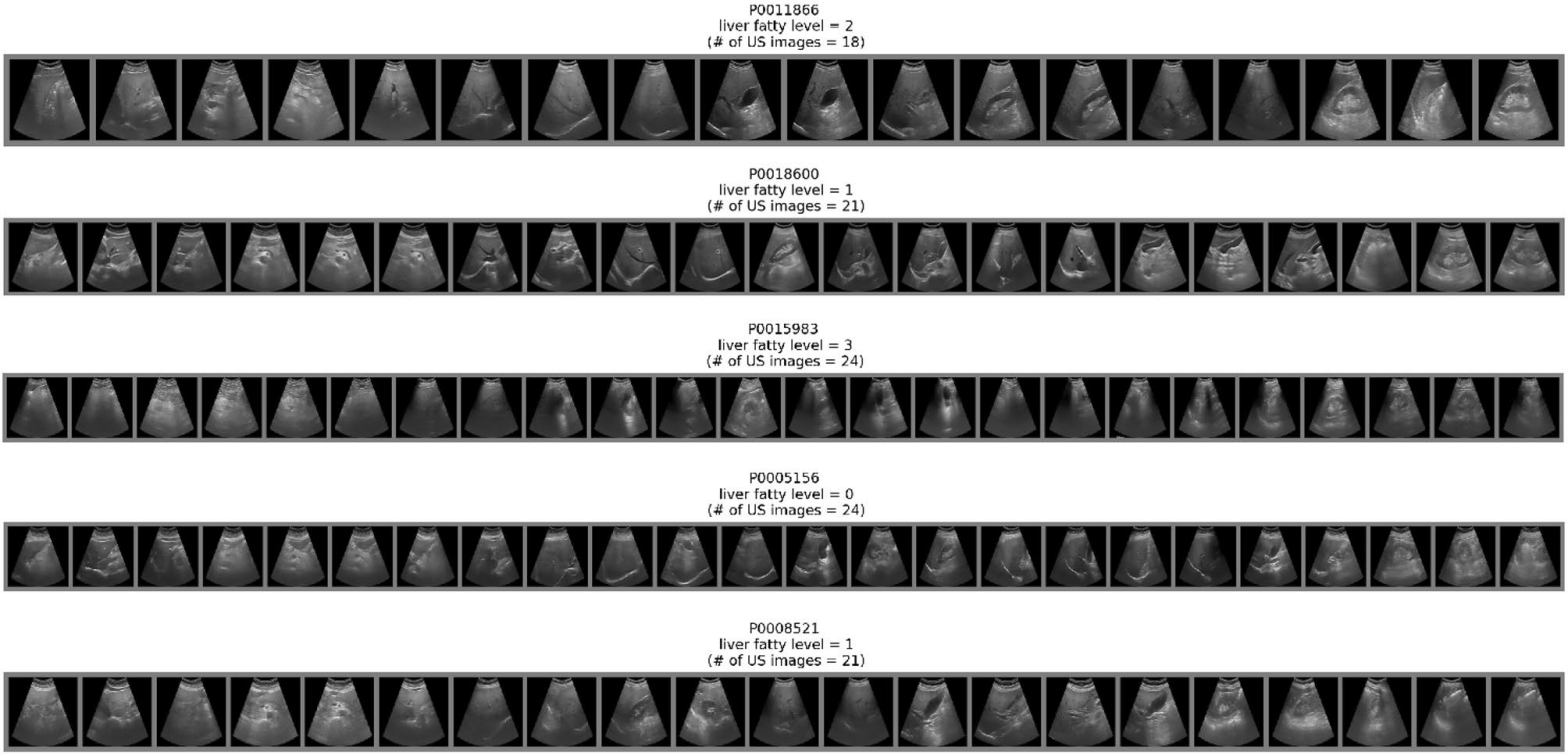
Examples of grouped ultrasound images in the TWBABD dataset.

It is known that there are accuracy limitations for diagnoses of fatty liver made by human physicians based on ultrasound images^2–4^. An alternative way is to deliver the diagnosis under a computer-aid framework. Recently machine learning has become a popular approach to computer-aid diagnosis with ultrasound imaging technologies^5,6^. Several studies have already focused on building machine learning models from ultrasound image data to deliver diagnoses of liver-related diseases such as liver cancer^7^ and fatty liver^8–15^. Most of the studies focused on training models for disease identification based on a single image from the patient. However, for organs such as the liver, ultrasound images usually have low resolutions. It is due to the inherent limitations of the scanning mechanism. As the liver is deep inside the body, low frequency pulses are required to obtain its ultrasound images. In addition, these images may also be obscured by bone, air or gases inside the body. To ensure a successful diagnosis on fatty liver, a patient may be required to take several images of the liver. These images are usually taken contingently, and different patients may take different numbers of ultrasound images, depending on requirements from the physician and the radiologist. Figure 1 shows examples of ultrasound images taken from five different patients. Each of them has different number of images. In real-world practice, the physician delivers a diagnostic decision to a patient by looking at not only one but also other images taken from the patient. The decision is a result based on several images. Some recent studies^16–18^ have tried to train models for disease classification based on grouped images from a single patient. However, models from these studies mainly treated grouped images as independent images. They first delivered an independent decision to each image, and then applied post-processing mechanisms to obtain a summarized decision from those independent decisions. Such a procedure might cause some interpretation issue. Since the grouped images may contain images of different organs, if the model’s classification rule only had fatty liver-specific classes, it might classify an image of a non-liver organ to a certain fatty liver type. In turn, this single decision might mislead the post-processing mechanisms to deliver a biased diagnosis. Moreover, when training these models, all images in the training dataset should be labeled. If the training dataset did not satisfy this condition, it would be difficult to train the models.

In this paper we propose a machine learning model that can deliver fatty liver diagnosis to a subject by looking at grouped ultrasound images from the subject. Unlike previous studies, our model does not use post-processing mechanisms to obtain a summarized decision from independent single decisions on each image. Instead, our model delivers a diagnostic decision to the patient by summarizing information from the abstract representation of each image. Because our model only delivers subject-level diagnosis, our training dataset only requires subject-level labeling. It does not require that all images should be labeled.

## Methods

In this section we first describe a machine learning model for fatty liver diagnosis based on grouped image data. We also describe a statistical method for controlling risk of the machine learning model based on the conformal prediction approach. We then describe the experiments we conducted for evaluating the machine learning model.

Since the experiments involved using ultrasound images from human subjects, we sent a proposal on the experiments to the Taiwan Biobank and the Institutional Review Board at Academia Sinica for review. Here we confirmed that all experiments in this paper have been approved by the Institutional Review Board at Academia Sinica with license numbers AS-IRB01-17049 and AS-IRB01-21009, and have been conducted under consent from all subjects involved in the experiments via the Institutional Review Board at Academia Sinica with license numbers AS-IRB01-17049 and AS-IRB01-21009. The waiver of informed consent is granted by the Institutional Review Board at Academia Sinica. All experiments were carried out in accordance with relevant guidelines and regulations under the Institutional Review Board at Academia Sinica and the Taiwanese government. All datasets used in the experiments are publicly available and can be downloaded via application at the Taiwan Biobank website https://www.biobank.org.tw/english.php.

### The machine learning model

Let ${{\textbf {x}}}=\{{{\textbf {x}}}_{1},{{\textbf {x}}}_{2},\ldots ,{{\textbf {x}}}_{S}\}$ denote the group of ultrasound images collected from a subject. The machine learning model aims to give a fatty liver diagnosis to the subject by using information from the grouped ultrasound images ${{\textbf {x}}}$. The model consists of two parts: an image encoder, which aims to extract features from each image in ${{\textbf {x}}}$; and an aggregate encoder, which aims to yield information for the diagnosis by summarizing the image features. Mathematically we can express the model as:

$$\begin{aligned} {{\textbf {h}}}_{s}= & {} \varvec{\phi }({{\textbf {x}}}_{s})\text { for }s=1,2,\ldots ,S,\\ \mathcal {G}= & {} G({{\textbf {h}}}_{1},{{\textbf {h}}}_{2},\ldots ,{{\textbf {h}}}_{S}),\\ ({{\textbf {u}}}_{1},{{\textbf {u}}}_{2},\ldots ,{{\textbf {u}}}_{S})= & {} \varvec{\psi }({{\textbf {h}}}_{1},{{\textbf {h}}}_{2},\ldots ,{{\textbf {h}}}_{S},\mathcal {G}),\\ {{\textbf {z}}}= & {} \varvec{\theta }({{\textbf {u}}}_{1},{{\textbf {u}}}_{2},\ldots ,{{\textbf {u}}}_{S}), \end{aligned}$$

where $\varvec{\phi }$ is the image encoder, ${{\textbf {h}}}_{s}$ is the feature of image $x_{s}$, *G* is a graph constructor, $\mathcal {G}$ is a graph in which the image features $\{{{\textbf {h}}}_{1},{{\textbf {h}}}_{2},\ldots ,{{\textbf {h}}}_{S}\}$ are attributes of the nodes, $\varvec{\psi }$ is a graph encoder, $\varvec{\theta }$ is an aggregate layer, and ${{\textbf {z}}}$ is a summary of the features $\{{{\textbf {u}}}_{1},{{\textbf {u}}}_{2},\ldots ,{{\textbf {u}}}_{S}\}$ processed by the graph encoder $\varvec{\psi }$. With ${{\textbf {z}}}$ we can build an output layer for the diagnostic decision. If there are *K* types of fatty liver diagnoses, then the output layer can be expressed as

$$\begin{aligned} \widehat{k}=\arg \max _{k\in \{1,2,\ldots ,K\}}\{\sigma _{1}({{\textbf {z}}}),\sigma _{2}({{\textbf {z}}}),\ldots ,\sigma _{K}({{\textbf {z}}})\}, \end{aligned}$$

where

$$\begin{aligned} \sigma _{k}({{\textbf {z}}})=\frac{\exp (\delta _{k}+\varvec{\beta }_{k}^{\top }{{\textbf {z}}})}{\sum _{k^{\prime }=1}^{K}\exp (\delta _{k^{\prime }}+\varvec{\beta }_{k^{\prime }}^{\top }{{\textbf {z}}})}. \end{aligned}$$

**Figure 2:**
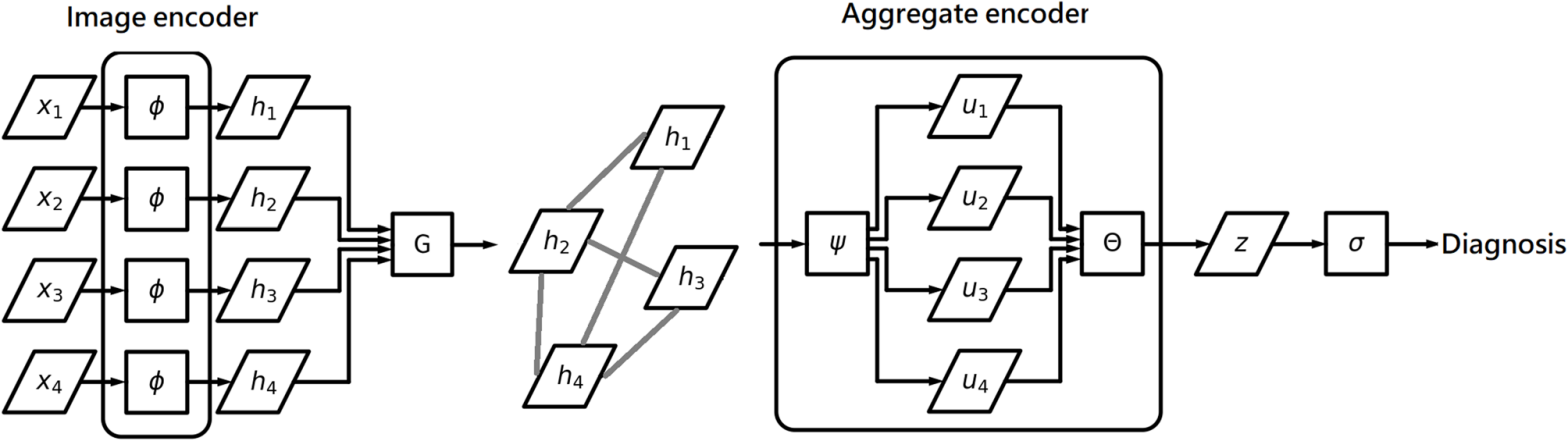
The deep neural network architecture for fatty liver diagnosis from grouped ultrasound images.

Here $\widehat{k}$ is the diagnostic decision, and $\sigma _{k}({{\textbf {z}}})$ is a score of diagnosis *k* conditional on summary information ${{\textbf {z}}}$, and $(\delta _{k},\varvec{\beta }_{k})$ is a parameter vector corresponding to diagnosis *k*. Figure 2 shows the schematic plot of the model.

#### Deep neural network architectures

There are several choices for the image encoder $\varvec{\phi }$. In this paper we consider the following deep neural network architectures: the Residual Neural Network (ResNet50^19^), the Densely Connected Convolutional Network (DenseNet121^20^), and Vision Transformer (ViT^21^), which is based on the Transformer models that are widely used in natural language processing^22,23^.

The aggregate encoder consists of two parts: the graph encoder $\varvec{\psi }$ and the aggregate layer $\varvec{\theta }$. The graph encoder $\varvec{\psi }$ should be invariant to permutations of the image features $\{{{\textbf {h}}}_{1},{{\textbf {h}}}_{2},\ldots ,{{\textbf {h}}}_{S}\}$. This means the diagnostic decision made by the deep neural network should not change if the ultrasound images $\{{{\textbf {x}}}_{1},{{\textbf {x}}}_{2},\ldots ,{{\textbf {x}}}_{S}\}$ are scanned in different orders^24,25^. In this paper we mainly consider the following graph neural networks for building the graph encoder: Graph Attention Network (GAT^26^) and Graph Isomorphism Network (GIN^27^). For the aggregate layer $\varvec{\theta }$, we adopt a global pooling layer based on the max function. For more details of the graph encoder $\varvec{\psi }$ and the aggregate layer $\varvec{\theta }$, please see Appendix A in Supplemental Materials. In addition, the graph encoder $\varvec{\psi }$ needs a graph $\mathcal {G}$ for carrying out forward propagation. The graph $\mathcal {G}$ has the image features $\{{{\textbf {h}}}_{1},{{\textbf {h}}}_{2},\ldots ,{{\textbf {h}}}_{S}\}$ as the attributes of the nodes. It is constructed by an operator *G* in which the link between two nodes *s* and *r* is defined by $e(s,r) = 1$ if Corr$({{\textbf {h}}}_{s},{{\textbf {h}}}_{r})\ge 0.995$, and $e(s,r) = 0$ otherwise, where Corr$({{\textbf {h}}}_{s},{{\textbf {h}}}_{r})$ is the Pearson correlation between image features ${{\textbf {h}}}_{s}$ and ${{\textbf {h}}}_{r}$.

#### Conformal prediction

In practice it is important to take the diagnostic decision under a risk control framework so that the physicians can know how much probability the decision might be wrong. The risk control should serve as a precaution measure for the physician. With such a precaution measure, the physician can formulate a proper response to the wrong decision and therefore reducing the damage caused by the decision.

Below we introduce conformal prediction^28,29^, an approach to controlling the risk of a machine learning model by providing a confidence set for the model’s output. In a classification task, this confidence set is also called the prediction set. Under conformal prediction, the prediction set can be constructed via a two-stage procedure. At the first stage, we compute a specified statistic for each sample in the calibration dataset. This specified statistic is called the conformity score. It measures the minimal size of the prediction set provided by the machine learning model to cover the correct label of the sample. With these conformity scores, we can establish an empirical distribution. At the second stage, we first set a risk control level and then select a value from the empirical distribution according to the risk control level. This value is selected to construct the prediction set so that the prediction set has high probability to cover the correct label.

In this paper we mainly consider two conformal prediction methods for controlling the risk of the machine learning models. The first method is called the Adaptive Prediction Sets (APS^30^), and the second method is called the Regularized Adaptive Prediction Sets (RAPS^31^). Both of the methods are designed for dealing with classification problems. Although both of the methods apply a randomized mechanism to compute conformity scores, RAPS is different from the APS in that RAPS adds a penalty term to the conformity score for punishing the model if the model includes too many undesirable labels in the prediction set. For more details, please see Appendix B in Supplemental Materials.

### Experiments

Below we conducted experiments for evaluating our machine learning model. Before presenting the experiment results, we describe settings in these experiments. These settings include the dataset, learning tasks, the training procedure and evaluation metrics.

#### TWBABD dataset

**Figure 3:**
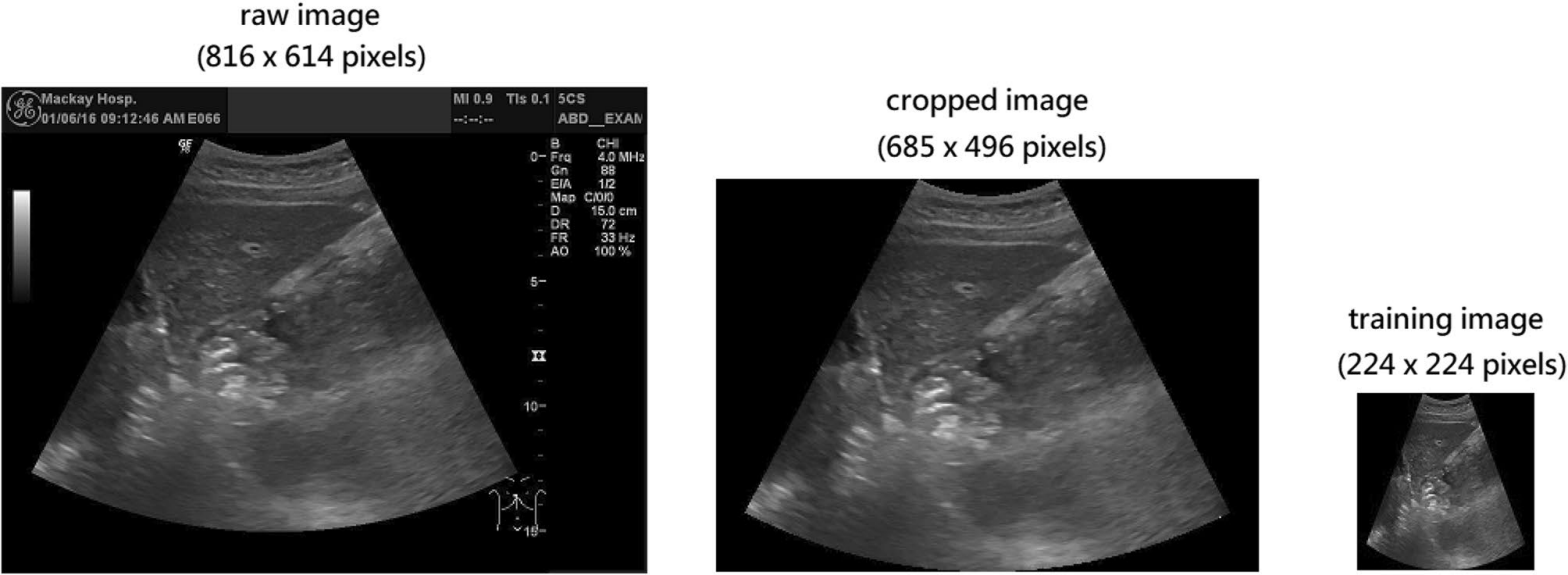
Ultrasound images from the Taiwan Biobank.

The ultrasound image dataset we used to train our machine learning model is provided by Taiwan Biobank (TWB). Taiwan Biobank is a large database that consists of nearly 200,000 participants of Taiwanese population. It is considered as one of the largest biobanks in Asia. At the early stage it aimed to collect phenotypic and genetic data for bio-medical investigation. Since 2020, it has also started to collect medical image data, including electrocardiographs, bone mass measurements, and ultrasound images. Currently there are 21,636 participants who have completed the abdominal ultrasound image scanning. The 21,636 participants are the subjects of the dataset in our study. The scanning tasks of the 21,636 subjects were all conducted at the Mackay Memorial Hospital Health Examination Center in Taipei. The resulting ultrasound images were stored in the Digital Imaging and Communications in Medicine (DICOM) format before being examined and labeled by radiographers and physicians. In practice, we pre-processed these ultrasound images to remove the device marker and then stored these images in the PNG format for further model training purposes. Figure 3 shows the pre-processing procedure.

In our dataset, each subject is labeled as one of the following four disease types: normal status, mild fatty liver, moderate fatty liver, and severe fatty liver. For quality control purposes, some subjects are also given “uncertainty” labels to indicate whether or not their ultrasound images are difficult to examine. To make our dataset cleaner, we further conducted a quality control procedure to exclude subjects with such uncertain labels. The resulting dataset contains abdominal ultrasound images corresponding to 16,772 subjects. Among the 16,772 subjects, 12,321 are in the normal status, 2287 are labeled as mild fatty liver, 1629 are labeled as moderate fatty liver, and 535 are labeled as severe fatty liver. For more details, please see Table 1 and Appendix C in Supplemental Materials.

**Table 1:** The numbers of subjects of the four disease types in the TWB ultrasound image dataset.

Diagnosis	Normal	Mild	Moderate	Severe	Total
Number of subjects	12,321	2,287	1,629	535	16,772
Proportion	73.5%	13.6%	9.7%	3.2%	100%

#### Tasks

We aim to train our machine learning model by solving three classification tasks. The first task is a two-class classification task, in which the machine learning model is asked to identify whether a subject is normal or is in the disease status. The disease status includes all three fatty liver types in the dataset: mild fatty liver, moderate fatty liver, and severe fatty liver. The second task is a three-class classification task. In this task, labels of the moderate fatty liver and severe fatty liver are combined into one class. The machine learning model is asked to identify whether a subject is normal, or has mild fatty liver, or has either moderate or severe fatty liver. The third task is a four-class classification task, in which the machine learning model is asked to identify whether a subject belongs to one of the following four disease types: normal, mild fatty liver, moderate fatty liver and severe fatty liver.

#### Model training

To train the machine learning model, our dataset was further divided into training, validation and test sets. The proportions of sizes for the training, validation, and test sets were fixed at 81%, 9% and 10%, respectively. In addition, a random down-sampling approach was adopted to balance the numbers of subjects in each disease type in the training, validation and test sets according to the tasks. Table 2 summarizes the numbers of subjects in the training, validation, and test sets for the three machine learning tasks. For example, for the four-class classification task there are 216 subjects in the test set, which means there are $216/4 = 54$ subjects in each class. This number is roughly 10% of the 535 severe fatty liver subjects in our dataset (see Table 1). Similar calculations can be done for other tasks.

**Table 2:** Sizes of training, validation, and test datasets for the three tasks.

Task	Training	Validation	Test
Two-class classification	7210	802	890
Three-class classification	5259	585	648
Four-class classification	1728	196	216

In practice, the following deep neural network architectures were adopted as the image encoder to build our machine learning model: (1) DenseNet121^20^, (2) ResNet50^19^, and (3) ViT-16, i.e. Vision Transformer^21^. In addition, the following deep neural network architectures are adopted as the graph encoder in our model: (1) the Graph Attention Network (GAT^26^) with one layer block, (2) the Graph Attention Network with two layer blocks, (3) the Graph Isomorphism Network (GIN^27^) with one layer block, and (4) the Graph Isomorphism Network with two layer blocks. Details of these deep neural network architectures can be found in Appendix A in Supplemental Materials.

When training these deep neural network architectures, we first pre-trained the image encoders on publicly available datasets. DenseNet121 and ResNet50 were pre-trained on the ImageNet dataset while Vision Transformer was pre-trained on ImageNet-21k dataset. We then applied the pre-trained image encoders to the training data to obtain the their image features. For DenseNet121, ResNet50, and Vision Transformer, the corresponding dimensions of the image features are 1024, 2048, and 1024, respectively. These image features were then used to construct the graph for training the graph encoders. We set the inner (hidden) dimensions of all graph encoders equal to 512. We used the cross entropy as the loss function. To run the training procedure, we set the batch size of the training data equal to 32 and the number of training epochs equal to 100. We run the training procedure by using the ADAM algorithm^32^ with Cosine-Annealing^33^ for the learning rate adjustment.

#### Model evaluation

We visually evaluated performance of the machine learning models by drawing the confusion matrix and ROC curves for the results. We also evaluated overall performance of the model by using the following metrics. The first metric is accuracy, which is defined by

$$\begin{aligned} \text {accuracy}=\frac{\#\text { of correctly classified subjects}}{\#\text { of subjects}}. \end{aligned}$$

To evaluate class-specific performance, the following metrics were considered: (1) precision, which is defined by

$$\begin{aligned} \text {precision}=\frac{\text {TP}}{\text {TP}+\text {FP}}, \end{aligned}$$

where TP is the number of subjects who are correctly classified to the class, FP is the number of subjects who are wrongly classified to the class; (2) recall, which is defined by

$$\begin{aligned} \text {recall}=\frac{\text {TP}}{\text {TP}+\text {FN}}, \end{aligned}$$

where FN is the number of subjects who belong to the class but are wrongly classified to other classes; and (3) f1-score, which is defined by

$$\begin{aligned} \text {f1-score}=\frac{\text {TP}}{\text {TP}+0.5(\text {FP}+\text {FN})}, \end{aligned}$$

To evaluate conformal prediction, the following two metrics were adopted: the prediction set size and the coverage rate, which are defined by

$$\begin{aligned} \text {prediction set size}=\#\text { labels in the prediction set}, \end{aligned}$$

and

$$\begin{aligned} \text {coverage rate}=\frac{\#\text { of subjects who are correctly covered by the prediction set}}{\#\text { of subjects}}, \end{aligned}$$

respectively. Note that in the above formula when all the prediction set sizes are equal to 1, the coverage rate becomes the accuracy.

## Results

We conducted the experiments by applying the machine learning model to solve the following three tasks: (1) two-classification task involving normal and fatty liver cases; (2) three-classification task involving normal, mild fatty liver, and moderate$+$severe fatty liver cases; (3) four-class classification task involving normal, mild fatty liver, moderate fatty liver, severe fatty liver cases. Below we present results of the three experiments.

### Two-class classification

**Figure 4:**
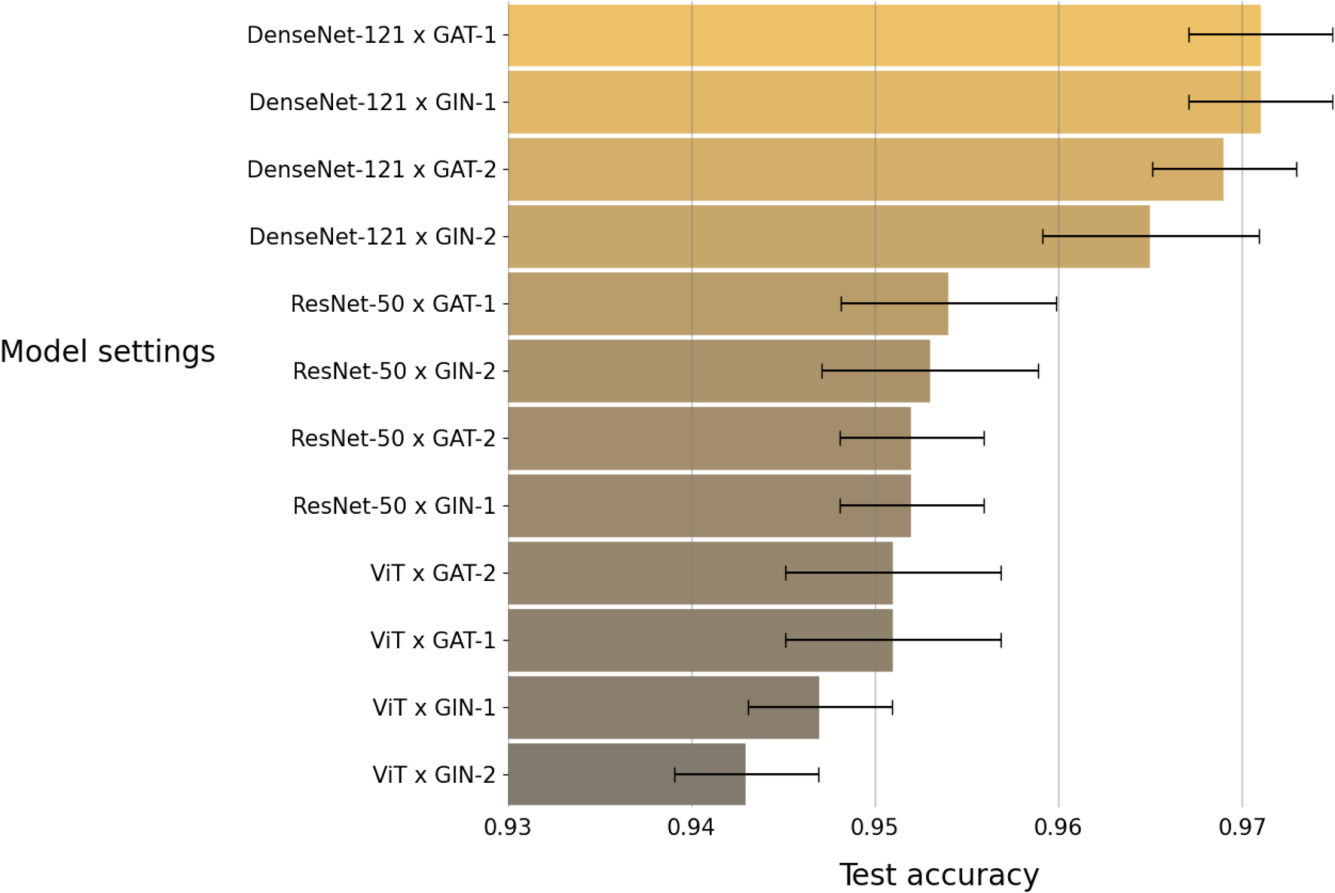
Accuracy for the two-class classification task by the 12 model settings. Each bar represents an average of accuracy over 10 repeated experiments.

**Figure 5:**
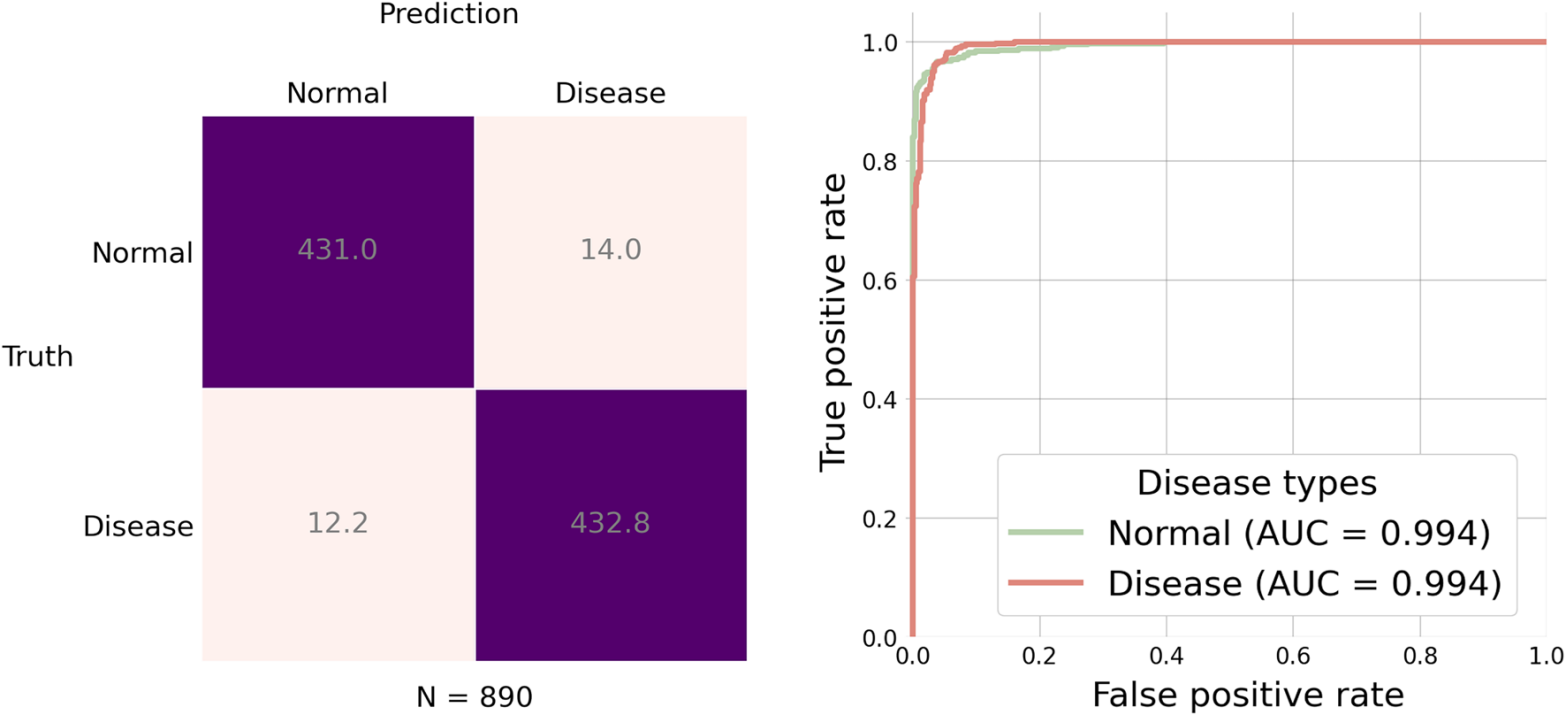
Results for the two-class classification task by the best model setting in Fig. 4. In the test set, each disease type has $n = 445$ subjects. Left: confusion matrix; Right: ROC curves.

This task aims to train a model that can identify whether a subject is in the normal condition or has fatty liver. We considered 12 different settings (3 image encoders $\times$ 4 graph/aggregate encoders) for the model. We trained the model on a training set with 7210 subjects, and then evaluated the model on a test set with 890 subjects. Both training and test sets were balanced, i.e. in the training set, each class has 3605 subjects, and in the test dataset, each class has 445 subjects. We repeated this training and test procedure 10 times on 10 different training and test sets. We evaluated the model by computing the performance measures described in “Model evaluation” section. Figure 4 shows accuracy of the model under the 12 different settings. We can see that the highest accuracy is provided by the model with DenseNet-121 as the image encoder and Graph Attention Network (GAT) with one block as the graph encoder. Figure 5 further shows the confusion matrix and the ROC curves of the results from the model with the best setting.

We further performed conformal prediction on the test set using the model with the best setting. We considered three conformal prediction methods: Naive Prediction Sets (NPS), Adaptive Prediction Sets (APS^30^), and Regularized Adaptive Prediction Sets (RAPS^31^). We set the risk level $\alpha$ equal to 0.1. This means that under this value a prediction set should cover the ground truth label with at least 90% probability. The threshold *q* was estimated from conformity scores of the validation set with 802 subjects. Table 3 shows the results of the conformal prediction. The RAPS procedure has the smallest average prediction set size, meaning that on average the RAPS procedure assigned 1.046 disease types to each subject. For those in the unambiguous group, i.e. subjects assigned with only one disease type, the coverage rate (i.e. the accuracy) of the RAPS procedure is 98.4%.

**Table 3:** Results of conformal prediction for the two-class classification task by the model with the best setting in Fig. 4. The value in the bracket is the corresponding standard error.

Method	Prediction set size	Coverage rate (unambiguous)	Coverage rate (ambiguous)	Coverage rate (all)
NPS	1.088 (0.005)	0.993 (0.001)	1.000 (0.000)	0.993 (0.001)
APS	1.962 (0.011)	1.000 (0.000)	1.000 (0.000)	1.000 (0.000)
RAPS	1.046 (0.003)	0.984 (0.001)	1.000 (0.000)	0.985 (0.000)

We also evaluated performance of the RAPS procedure by comparing its results with the results without conformal prediction. Table 4 shows the values of precision, recall and f1-score of each of the two disease types for the model with the best setting with conformal prediction (DenseNet-121 $\times$ GAT-1 with RAPS) and without conformal prediction (DenseNet-121 $\times$ GAT-1). Note that the number of subjects in conformal prediction is 849.4, which is the average size of the unambiguous groups over 10 repeated experiments. This means that there are less than 5% ($890-849.4=40.6$) of the subjects in the test set belong to the ambiguous group, i.e. the group of subjects who are assigned with multiple disease types by the conformal prediction. In summary, the conformal prediction improves the performance of the model with the best setting in the two-class classification task.

**Table 4:** Comparisons between the model with the best setting in Fig. 4 with conformal prediction (top; $n=849.4$) and without conformal prediction (bottom; $n=890$). Each value represents an average over 10 repeated experiments. The value in the bracket is the corresponding standard error.

Model	Disease type	Precision	Recall	F1-score
Model with RAPS	Normal	**0.988 (0.002)**	**0.980 (0.002)**	**0.984 (0.001)**
	Disease	**0.980 (0.002)**	**0.988 (0.002)**	**0.984 (0.001)**
Model without RAPS	Normal	0.973 (0.003)	0.969 (0.002)	0.971 (0.002)
	Disease	0.969 (0.002)	0.973 (0.003)	0.971 (0.002)

The bold values refer to the better values between two models (Model with RAPS and Model without RAPS).

### Three-class classification

**Figure 6:**
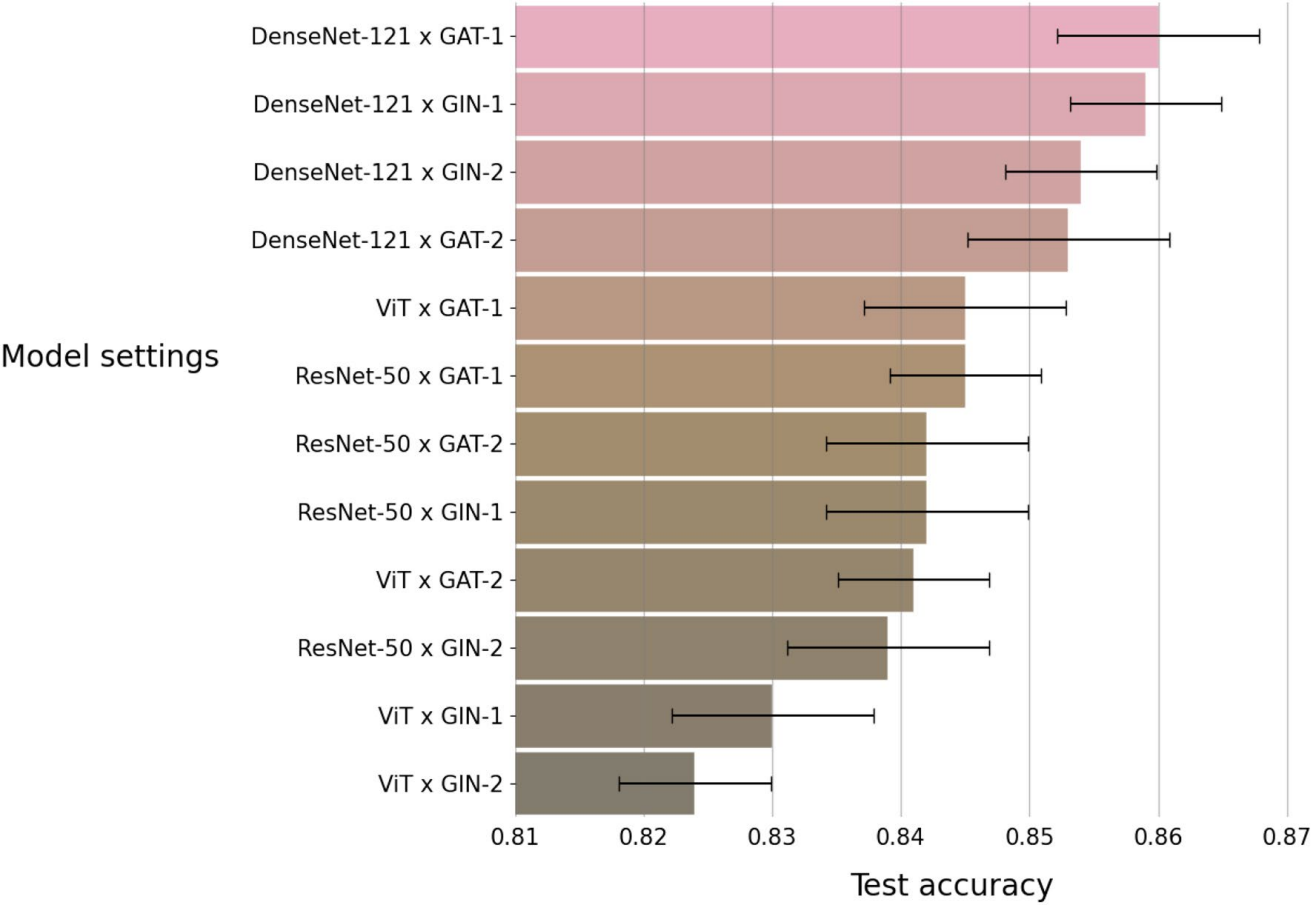
Accuracy for the three-class classification task by the 12 different model settings. Each bar represents an average of accuracy over 10 replicates.

**Figure 7:**
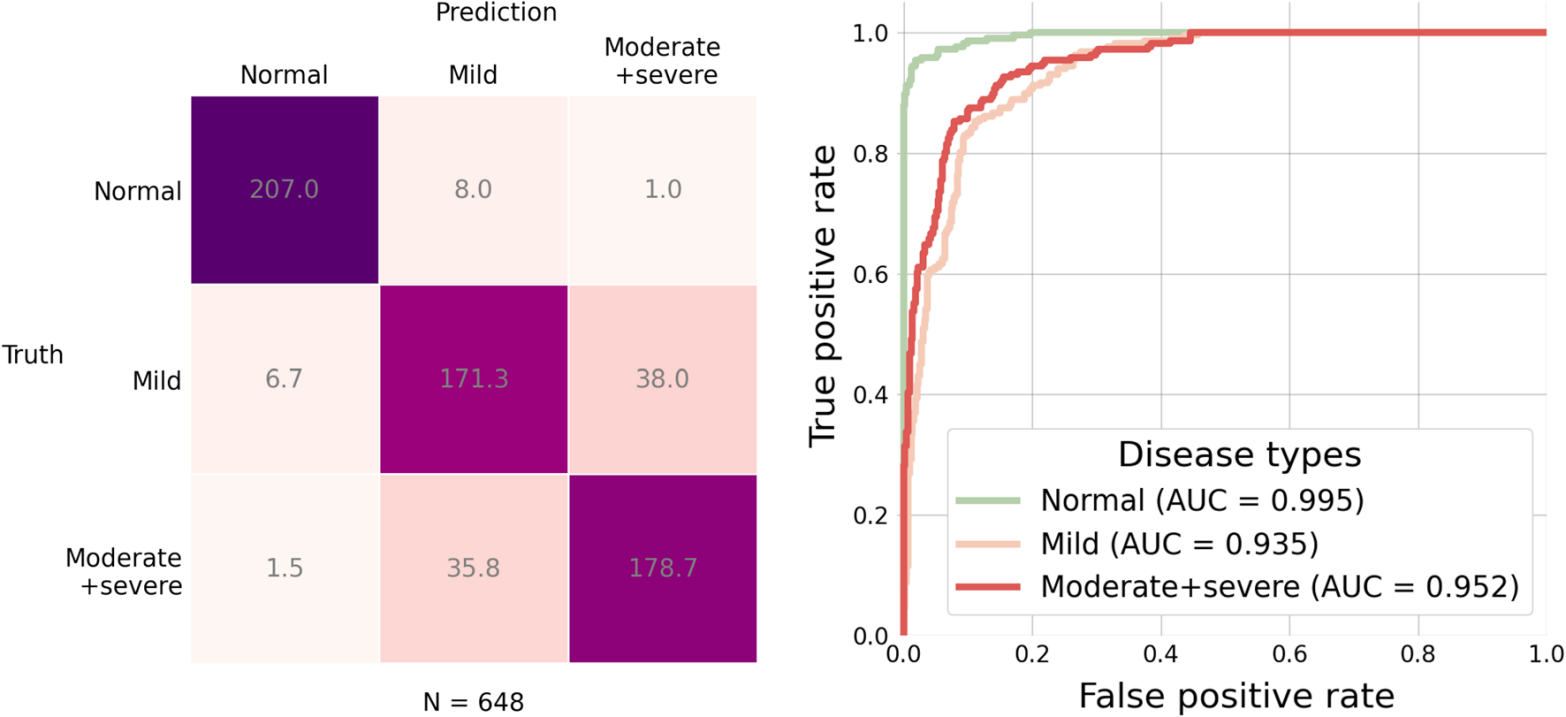
Results for the three-class classification task by the model with the best setting in Figure 6. Each class has $n = 216$ subjects in the test set. Left: confusion matrix; Right: ROC curves.

The three-class classification task further considers two sub-types of fatty liver. The two sub-types are mild fatty liver and moderate/severe fatty liver. We trained the model with 12 different settings by applying the training and test procedure the same as the one in the two-class classification task. Figure 6 shows accuracy of the 12 different model settings. We can see that the highest accuracy is provided by the model with DenseNet-121 as the image encoder and Graph Attention Network (GAT) with one block as the graph encoder. Figure 7 further shows the confusion matrix and the ROC curves of the results from the model with the best setting.

We further performed conformal prediction on the test set with the model with the best setting. The risk level $\alpha$ was set to be 0.1, and the threshold *q* was estimated from conformity scores of 585 subjects in the validation set. Table 5 shows the results of the conformal prediction. The Regularized Adaptive Prediction Sets (RAPS) procedure has the smallest average prediction set size. On average it assigned 1.293 disease types to each subject. For those in the unambiguous group, i.e. subjects assigned with only one disease type, the coverage rate (i.e. the accuracy) of the RAPS procedure is 93.1%. For those in the ambiguous group, i.e. subjects assigned with multiple disease types, the coverage rate is 98.1%, which means that only less than 2% of the subjects in the ambiguous group have been assigned with wrong disease types.

**Table 5:** Results of conformal prediction for the three-class classification task by the best setting in Fig. 6. The value in the bracket is the corresponding standard error.

Method	Prediction set size	Coverage rate (unambiguous)	Coverage rate (ambiguous)	Coverage rate (all)
NPS	1.460 (0.010)	0.970 (0.003)	0.988 (0.002)	0.977 (0.002)
APS	2.522 (0.014)	1.000 (0.000)	1.000 (0.0)	1.000 (0.000)
RAPS	1.293 (0.011)	0.931 (0.003)	0.981 (0.004)	0.945 (0.003)

We also evaluated performance of the RAPS procedure by comparing its results with the results without conformal prediction. Table 6 shows the values of precision, recall and f1-score of each of the three disease types for the best model with conformal prediction (DenseNet-121 $\times$ GAT-1 with RAPS) and without conformal prediction (DenseNet-121 $\times$ GAT-1). Note that the number of subjects in conformal prediction is 465, which is the average size of the unambiguous groups over 10 repeated experiments. We can see the conformal prediction improves the performance of the best model on all three disease types.

**Table 6:** Comparisons between the model with best setting in Fig. 6 with conformal prediction (top; $n=465$) and without conformal prediction (bottom; $n=648$). Each value represents an average over 10 replicates. The value in the bracket is the corresponding standard error.

Model	Disease type	Precision	Recall	F1-score
Model with RAPS	Normal	**0.989 (0.001)**	**0.990 (0.002)**	**0.989 (0.001)**
	mild	**0.872 (0.004)**	**0.857 (0.015)**	**0.864 (0.008)**
	mod$+$sev	**0.904 (0.008)**	**0.911 (0.006)**	**0.907 (0.003)**
Model without RAPS	Normal	0.962 (0.003)	0.958 (0.003)	0.960 (0.002)
	Mild	0.797 (0.006)	0.793 (0.012)	0.794 (0.006)
	mod$+$sev	0.822 (0.009)	0.827 (0.007)	0.824 (0.004)

The bold values refer to the better values between two models (Model with RAPS and Model without RAPS).

### Four-class classification

**Figure 8:**
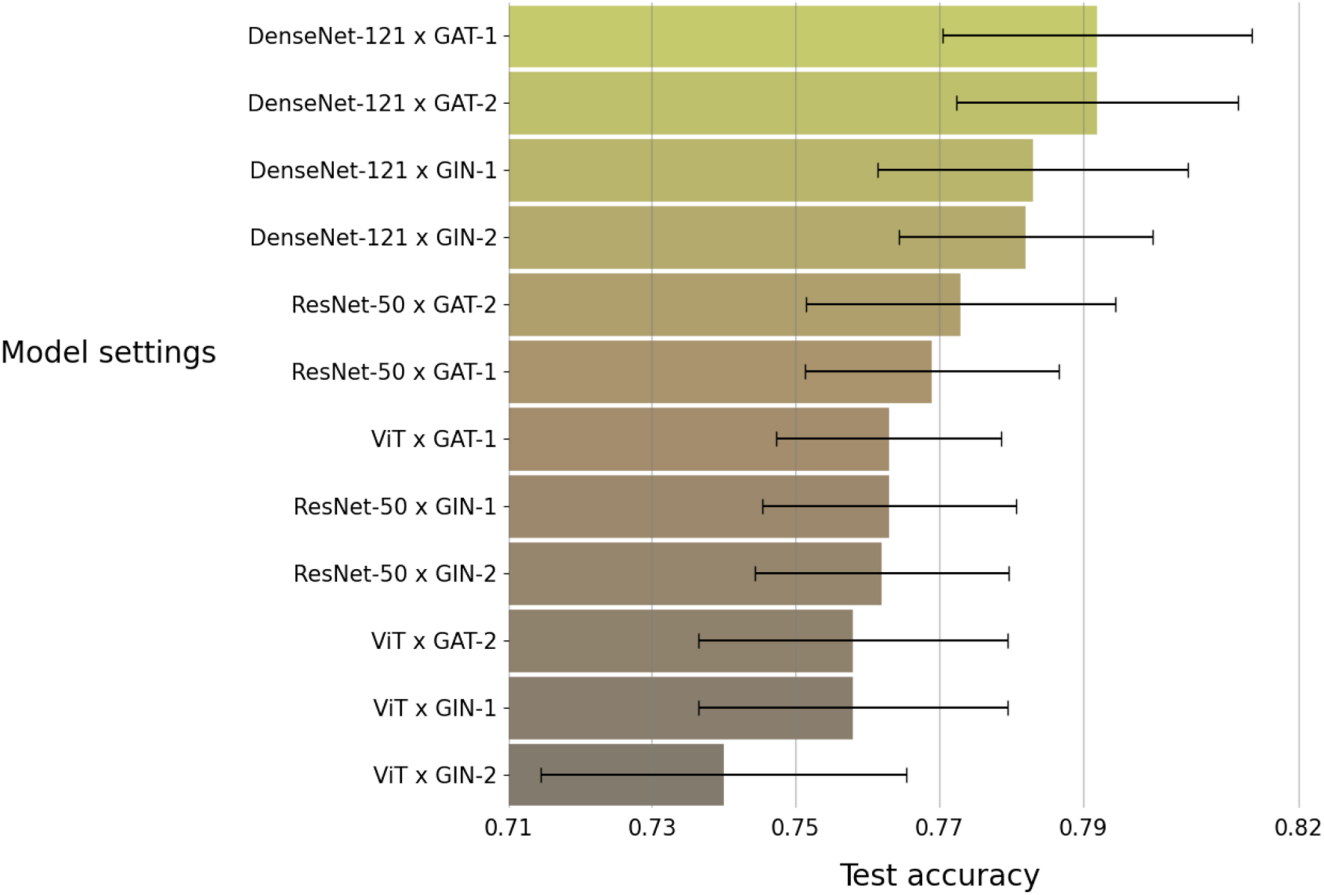
Accuracy for the four-class classification task by the 12 different settings. Each bar represents an average of accuracy over 10 repeated experiments.

**Figure 9:**
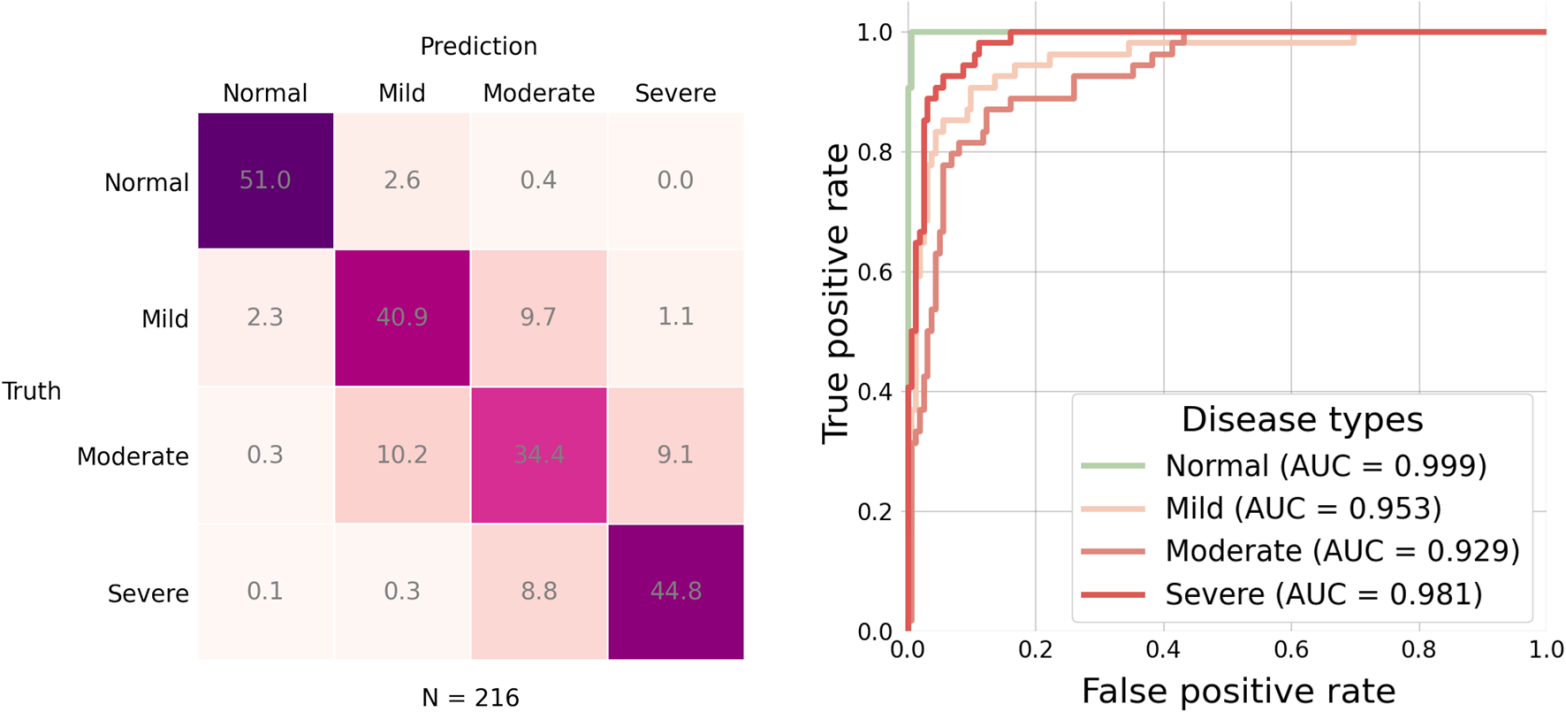
Results for the four-class classification task by the model with the best setting in Figure 8. In the test set, each disease type has $n = 54$ subjects. Left: confusion matrix; Right: ROC curves.

This task aims to train the machine learning model that can classify a subject to one of the following four disease types: the normal, mild fatty liver, moderate fatty liver, and severe fatty liver. In our training procedure, both training and test sets were balanced. The training set contains 1728 subjects, meaning that each class has 432 subjects. The test set contains 216 subjects, meaning that each class has 54 subjects. Figure 8 shows accuracy of the machine learning model with 12 different settings. We can see that the highest accuracy is provided by the model with DenseNet-121 as the image encoder and Graph Attention Network (GAT) with one block as the graph encoder. Figure 9 further shows the confusion matrix and the ROC curves of the model with the best setting.

We further performed conformal prediction on the test set using the model with the best setting. We set the risk level $\alpha$ equal to 0.1, and estimated the threshold *q* from conformity scores of the 196 subjects in the validation set. Table 7 shows the results of the conformal prediction. The RAPS procedure has the smallest average prediction set size equal to 1.525, which means that on average the RAPS procedure assigned 1.525 disease types to each subject. For those in the unambiguous group, i.e. subjects assigned with only one disease type, the coverage rate (i.e. the accuracy) of the RAPS procedure is 92.2%. For those in the ambiguous group, i.e. subjects assigned with multiple disease types, the coverage rate is 96.0%, meaning that only 4% of the subjects in the ambiguous group have been assigned with wrong disease types.

**Table 7:** Results of conformal prediction for the four-class classification task by the model with the best setting in Fig. 8. The value in the bracket is the corresponding standard error.

Method	Prediction set size	Coverage rate (unambiguous)	Coverage rate (ambiguous)	Coverage rate (all)
NPS	1.740 (0.018)	0.972 (0.004)	0.974 (0.005)	0.973 (0.003)
APS	3.035 (0.045)	1.0 (0.0)	0.999 (0.001)	0.999 (0.001)
RAPS	1.525 (0.018)	0.922 (0.006)	0.960 (0.006)	0.940 (0.004)

We also evaluated performance of the RAPS procedure by comparing its results with the results without conformal prediction. Table 8 shows the values of precision, recall and f1-score of each of the four classes for the best model with conformal prediction (DenseNet-121 $\times$ GAT-1 with RAPS) and without conformal prediction (DenseNet-121 $\times$ GAT-1). Note that the number of subjects in conformal prediction is 115, which is the average size of the unambiguous groups over 10 repeated experiments. We can see the conformal prediction improves the performance of the best model on three of the four disease types.

**Table 8:** Comparisons between the model with the best setting in Fig. 8 with conformal prediction (top; $n=115$) and without conformal prediction (bottom; $n=216$). Each value represents an average over 10 repeated experiments. The value in the bracket is the corresponding standard error.

Model	Class	Precision	Recall	F1-score
Model with RAPS	Normal	**0.994 (0.003)**	**0.996 (0.003)**	**0.995 (0.002)**
	Mild	**0.878 (0.013)**	**0.949 (0.017)**	**0.911 (0.012)**
	Moderate	0.589 (0.065)	0.368 (0.052)	0.445 (0.055)
	Severe	**0.902 (0.013)**	**0.955 (0.012)**	**0.927 (0.010)**
Model without RAPS	normal	0.956 (0.007)	0.956 (0.010)	0.955 (0.005)
	Mild	0.777 (0.015)	0.778 (0.016)	0.776 (0.010)
	Moderate	**0.652 (0.016)**	**0.643 (0.020)**	**0.646 (0.015)**
	Severe	0.819 (0.011)	0.822 (0.016)	0.820 (0.010)

The bold values refer to the better values between two models (Model with RAPS and Model without RAPS).

### Ablation study

We conducted ablation study on the machine learning model by considering three different encoders that do not exploit graph structure for feature encoding. The three encoders are based on the following neural network architectures: (a) a multilayer perceptron with one layer block (MLP-1), (b) a multiplayer perceprtron with two layer blocks (MLP-2), and (c) a global pooling layer based on the max function (MAX), respectively. We evaluated performance of the machine learning model with the three different encoders on solving the three classification tasks by following experiment settings the same as those used in the previous sections. We compared their performances to the performance of the best setting (DenseNet-121$\times$GAT-1) in terms of accuracy and reported the results in Table 9. From Table 9 we can see DenseNet-121$\times$GAT-1, the only setting in the table that explores graph of image features for forward propagation, has the highest accuracy values in all three classification tasks. The results suggest that the graph encoder, i.e. Graph Attention Network (GAT), outperforms other encoders that do not account for the graph of image features, highlighting the importance of considering such graph in encoding grouped images.

**Table 9:** Performance of the machine learning model under different settings for solving the three classification tasks. The value is the accuracy calculated over 10 replicates. The value in the bracket is the corresponding standard error. MLP-1 refers to a multilayer perceptron with one layer block; MLP-2 refers to a multiplayer perceprtron with two layer blocks; and MAX refers to a global pooling layer based on the max function.

Model	Two-class classification	Three-class classification	Four-class classification
DenseNet-121$\times$GAT-1	**0.971** (0.002)	**0.860** (0.004)	**0.792** (0.011)
DenseNet-121$\times$MLP-2	0.959 (0.002)	0.845 (0.004)	0.778 (0.009)
DenseNet-121$\times$MLP-1	0.963 (0.003)	0.855 (0.004)	**0.792** (0.008)
DenseNet-121$\times$MAX	0.897 (0.003)	0.792 (0.006)	0.709 (0.005)
ViT$\times$MLP-2	0.946 (0.003)	0.836 (0.003)	0.764 (0.007)
ViT$\times$MLP-1	0.948 (0.002)	0.844 (0.003)	0.773 (0.008)
ViT$\times$MAX	0.916 (0.003)	0.813 (0.004)	0.738 (0.008)
ResNet-50$\times$MLP-2	0.944 (0.004)	0.832 (0.004)	0.761 (0.008)
ResNet-50$\times$MLP-1	0.945 (0.003)	0.836 (0.003)	0.769 (0.009)
ResNet-50$\times$MAX	0.912 (0.003)	0.802 (0.004)	0.711 (0.007)

The bold values refer to the better values between two models (Model with RAPS and Model without RAPS).

### Discussion

As a diagnosis method, ultrasound imaging has several advantages. It has relatively low cost to operate. It is also relatively safe as its operation does not involve radiation exposure. Moreover, it is portable and can be operated quickly, allowing medical professionals to access real-time images. However, due to the inherent limitations of its scanning mechanism, ultrasound imaging also requires multiple shots of the target organs to ensure a successful scanning. This procedure yields grouped image data. In this paper we propose a machine learning model trained on the grouped image data for fatty liver classification. The machine learning model has a hybrid deep neural network architecture consisting of an image encoder for extracting features from grouped images and an aggregate encoder for summarizing features of the grouped images according to the graph structure behind those image features. The experiment results show that the machine learning model achieves 97.1% accuracy (AUC $=0.994$) in two-class classification task, and further achieves 98.4% accuracy when Regularized Adaptive Prediction Sets, a conformal prediction method, is used for controlling the risk of the results (Table 3). In addition, it also achieves 86.0% accuracy in three-class classification task, and further achieves 93.1% accuracy with the conformal prediction method (Table 5). Moreover, when carrying out four-class classification task, the machine learning model achieves 79.2% accuracy and 92.2% accuracy when the conformal prediction method is used (Table 7).

We also investigated efficiency of the machine learning model by carrying out ablation study on graph encoders. The results show that the machine learning model can achieve best performance in all three classification tasks when a graph encoder is used for feature summary. This implies that imposing graph structure on the grouped image data can improve the overall performance of the machine learning model.

There are several limitations in our study. Firstly, we did not evaluate the machine learning model on external data from other sources. The reason is that currently it is not easy to find publicly available datasets with aims, purposes, and data structure similar to the dataset we used for training our model.

Secondly, an issue we did not address in this paper is about the interpretability of the results provided by the machine learning model. It is due to the difficulty of generating the saliency map for grouped images at once. Due to this difficulty, we are unable to explain our results in a clear and reasonable way. In the future we are planning to address this issue via more plausible approaches, by following recently-developed explainable AI methods such as those based on Local Interpretable Model-agnostic Explanation^34^ and Shapley additive explanations^35^.

Finally, although the machine learning model achieves 79.2% accuracy in the four-class classification task, it also has difficulty in classifying subjects who are in the moderate fatty liver condition. This difficulty may be due to several reasons. Although the moderate condition has a clear definition in terms of measurable quantities, such quantities may be difficult to be detected by only looking at the ultrasound images. In addition, the machine learning model sees the four fatty liver conditions as four distinct classes. It does not impose any quantitative order among the four fatty liver conditions. Moreover, the data were labeled by humans. As a result of that the data were still subject to human errors.

In summary, we have presented a machine learning model for fatty liver classification. To train this model, we also have presented a dataset that consists of abdominal ultrasound image collected by Taiwan Biobank, one of the largest community-based biobanks in Asian populations. In this dataset each subject has a bundle of images sharing the same label at the subject level. It is contrast to previous studies^8–18^ in which each image in the training dataset has its own label. To fully utilizing these intra-individual correlated image data, the machine learning model relies on a hybrid neural network architecture, which firstly extracts features from these images and then organizes these features together by exploring their graph structure. The experimental results show that the machine learning model can achieve high accuracy in various classification tasks. To make the experimental results more robust, we also have presented a statistical procedure based on conformal prediction. This procedure serves to mitigate the risk of inaccurate results provided by the machine learning model, providing a formal statistical statement about the “confidence of the results”, and indicating under what probability the result may be incorrect. Under this statistical procedure, the machine learning model can further improve its performances in these classification tasks.

### Supplementary Information

Supplementary Information.

## Data Availability

No datasets were generated or analysed during the current study.

## References

[1] Li J, Zou B, Yeo YH, Feng Y, Xie X, Lee DH, Nguyen MH (2019). Prevalence, incidence, and outcome of non-alcoholic fatty liver disease in Asia, 1999–2019: A systematic review and meta-analysis. Lancet Gastroenterol. Hepatol..

[2] Hernaez R, Lazo M, Bonekamp S, Kamel I, Brancati FL, Guallar E, Clark JM (2011). Diagnostic accuracy and reliability of ultrasonography for the detection of fatty liver: A meta-analysis. Hepatology.

[3] Pirmoazen AM, Khurana A, Kaffas AE, Kamaya A (2020). Quantitative ultrasound approaches for diagnosis and monitoring hepatic steatosis in nonalcoholic fatty liver disease. Theranostics.

[4] Ballestri S, Mantovani A, Byrne CD, Lonardo A, Targher G (2021). Diagnostic accuracy of ultrasonography for the detection of hepatic steatosis: An updated metaanalysis of observational studies. Metab. Target Organ Damage.

[5] Avola D, Cinque L, Fagioli A, Foresti G, Mecca A (2021). Ultrasound medical imaging techniques: A survey. ACM Comput. Surv..

[6] Micucci M, Iula A (2022). Recent advances in machine learning applied to ultrasound imaging. Electronics.

[7] Yang Q, Wei J, Hao X, Kong D, Yu X, Jiang T, Xi J, Cai W, Luo Y, Jing X, Yang Y, Cheng Z, Wu J, Zhang H, Liao J, Zhou P, Song Y, Zhang Y, Han Z, Cheng W, Liang P (2020). Improving B-mode ultrasound diagnostic performance for focal liver lesions using deep learning: A multicentre study. EBioMedicine.

[8] Byra M, Styczynski G, Szmigielski C, Kalinowski P, Michałowski L, Paluszkiewicz R, Ziarkiewicz-Wróblewska B, Zieniewicz K, Sobieraj P, Nowicki A (2018). Transfer learning with deep convolutional neural network for liver steatosis assessment in ultrasound images. Int. J. Comput. Assist. Radiol. Surg..

[9] Cao W, An X, Cong L, Lyu C, Zhou Q, Guo R (2020). Application of deep learning in quantitative analysis of 2-dimensional ultrasound imaging of nonalcoholic fatty liver disease. J. Ultrasound Med..

[10] Chen J-R, Chao Y-P, Tsai Y-W, Chan H-J, Wan Y-L, Tai D-I, Tsui P-H (2020). Clinical value of information entropy compared with deep learning for ultrasound grading of Hepatic Steatosis. Entropy.

[11] Che H, Brown LG, Foran DJ, Nosher JL, Hacihaliloglu I (2021). Liver disease classification from ultrasound using multi-scale CNN. Int. J. Comput. Assist. Radiol. Surg..

[12] Zamanian H, Mostaar A, Azadeh P, Ahmadi M (2021). Implementation of combinational deep learning algorithm for non-alcoholic fatty liver classification in ultrasound images. J. Biomed. Phys. Eng..

[13] Rhyou S-Y, Yoo J-C (2021). Cascaded deep learning neural network for automated Liver Steatosis diagnosis using ultrasound images. Sensors.

[14] Wu, C.-H., Hung, C.-L., Lee, T.-Y., Wu, C.-Y. & Chu, W. C.-C. Fatty liver diagnosis using deep learning in ultrasound image. In *IEEE International Conference on Digital Health (ICDH)* (2022).

[15] Alshagathrh FM, Househ MS (2022). Artificial intelligence for detecting and quantifying fatty liver in ultrasound images: A systematic review. Bioengineering.

[16] Chou T-H, Yeh H-J, Chang C-C, Tang J-H, Kao W-Y, Su I-C, Li C-H, Chang W-H, Huang C-K, Sufriyana H, Su EC-Y (2021). Deep learning for abdominal ultrasound: A computer-aided diagnostic system for the severity of fatty liver. J. Chin. Med. Assoc..

[17] Kim T, Lee DH, Park E-K, Choi S (2021). Deep learning techniques for fatty liver using multi-view ultrasound images scanned by different scanners: Development and validation study. J. Med. Internet Res..

[18] Zhang H, Guo L, Wang D, Wang J, Bao L, Ying S, Xu H, Shi J (2021). Multi-source transfer learning via multi-kernel support vector machine plus for B-mode ultrasound-based computer-aided diagnosis of liver cancers. IEEE J. Biomed. Health Inform..

[19] He, K., Zhang, X., Ren, S. & Sun, J. Deep residual learning for image recognition. *CVPR*, 770–778 (2016).

[20] Huang, G., Liu, Z., van der Maaten, L., Weinberger, K. Q. Densely connected convolutional networks. *CVPR* (2017).

[21] Dosovitskiy, A., Beyer, L., Kolesnikov, A., Weissenborn, D., Zhai, X., Unterthiner, T., Dehghani, M., Minderer, M., Heigold, G., Gelly, S., Uszkoreit, J. & Houlsby, N. An image is worth $$16\times 16$$ words: Transformers for image recognition at scale. *ICLR* (2021).

[22] Vaswani, A., Shazeer, N., Parmar, N., Uszkoreit, J., Jones, L., Gomez, A. N. & Kaiser, L. Attention is all you need. *NeurIPS* (2017).

[23] Devlin, J., Chang, M.-W., Lee, K. & Toutanova, K. BERT: Pre-training of deep bidirectional transformers for language understanding. arXiv arXiv:1810.04805v2 (2019).

[24] Kipf, T. N. & Welling, M. Semi-supervised classification with graph convolutional networks. *ICLR* (2017).

[25] Hamilton, W. L., Ying, R. & Leskovec, J. Inductive representation learning on large graphs. *NIPS* (2017).

[26] Velic̆ković, P., Cucurull, G., Casanova, A., Romero, A., Lió, P. & Bengio, Y. Graph attention networks. *ICLR* (2018).

[27] Xu, K., Hu, W., Leskovec, J. & Jegelka, S. How powerful are graph neural networks? *ICLR* (2019).

[28] Vovk V, Gammerman A, Shafer G (2005). Algorithmic Learning in a Random World.

[29] Angelopoulos, A. N. & Bates, S. A gentle introduction to conformal rrediction and distribution-free uncertainty quantification. arXiv arXiv:2107.07511v3 (2022).

[30] Romano, Y., Sesia, M. & Candés, E. J. Classification with valid and adaptive coverage. *NeurIPS* (2020).

[31] Angelopoulos, A. N., Bates, S., Malik, J. & Jordan, M. I. Uncertainty sets for image classifiers using conformal prediction. *ICLR* (2021).

[32] Kingma, D. P. & Ba, J. L. Adam: A method for stochastic optimization. *ICLR* (2015).

[33] Loshchilov, I. & Hutter, F. SGDR: Stochastic gradient descent with warm restarts. *ICLR* (2017).

[34] Ribeiro, M. R., Singh, S. & Guestrin, C. “Why should I trust you?” Explaining the predictions of any classifier. *KDD* (2016)

[35] Lundberg, S. M. & Lee, S.-I. A unified approach to interpreting model predictions. *NeurIPS* (2017).

