# Supplementary material for: Fatty liver classification via risk controlled neural networks trained on grouped ultrasound image data
Source: Sci Rep. 2024 Mar 28;14:7345. doi: 10.1038/s41598-024-57386-3 (PMC10973492; doi:10.1038/s41598-024-57386-3)
Supplement: Supplementary file 1 — Supplementary Information. [file 41598_2024_57386_MOESM1_ESM.pdf]

Supplemental Materials

Appendix A

In our paper we have used the Graph Attention Network (GAT, [11]), Graph Isomorphism Network (GIN, [13]), Multilayer Perceptron (MLP) to build the graph encoder $\boldsymbol{\psi}$, and used a global pooling layer to build the aggregate layer $\boldsymbol{\theta}$. Let $\mathcal{N}_s$ denote the neighborhood of s. Below we describe the forward propagation of these neural network blocks:

- Graph attention network (GAT): The forward propagation of the representation of the node s from block l to block $(l+1)$ is given by

$$
\begin{aligned}
\mathbf{h}_s^{[l+1/6]} &= \sum_{r\in\mathcal{N}_s} w_{sr}^{[l+1]}\boldsymbol{\Gamma}_1^{[l+1]}\mathbf{h}_r^{[l]},\\
\mathbf{h}_s^{[l+2/6]} &= \boldsymbol{\gamma}_3^{[l+1]} + \boldsymbol{\Gamma}_2^{[l+1]}\mathbf{h}_s^{[l+1/6]},\\
\mathbf{h}_s^{[l+3/6]} &= \mathrm{BatchNorm}(\mathbf{h}_s^{[l+2/6]}),\\
\mathbf{h}_s^{[l+4/6]} &= \mathrm{ReLU}(\mathbf{h}_s^{[l+3/6]}),\\
\mathbf{h}_s^{[l+5/6]} &= \boldsymbol{\gamma}_5^{[l+1]} + \boldsymbol{\Gamma}_4^{[l+1]}\mathbf{h}_s^{[l+4/6]},\\
\mathbf{h}_s^{[l+1]} &= \mathrm{ReLU}(\mathbf{h}_s^{[l+5/6]}).
\end{aligned}
$$

  where the attention weight $w_{sr}^{[l+1]}$ is defined in terms of a softmax function.

- Graph Isomorphism Network (GIN): The forward propagation of the representation of the node s from block l to block $(l+1)$ is given by

$$
\begin{aligned}
\mathbf{h}_s^{[l+1/5]} &= \boldsymbol{\gamma}_2^{[l+1]} + \boldsymbol{\Gamma}_1^{[l+1]}\left((1+\epsilon)\mathbf{h}_s^{[l]} + \sum_{r\in\mathcal{N}_s}\mathbf{h}_r^{[l]}\right),\\
\mathbf{h}_s^{[l+2/5]} &= \mathrm{BatchNorm}(\mathbf{h}_s^{[l+1/5]}),\\
\mathbf{h}_s^{[l+3/5]} &= \mathrm{ReLU}(\mathbf{h}_s^{[l+2/5]}),\\
\mathbf{h}_s^{[l+4/5]} &= \boldsymbol{\gamma}_4^{[l+1]} + \boldsymbol{\Gamma}_3^{[l+1]}\mathbf{h}_s^{[l+3/5]},\\
\mathbf{h}_s^{[l+1]} &= \mathrm{ReLU}(\mathbf{h}_s^{[l+4/5]}).
\end{aligned}
$$

- Multilayer Perceptron (MLP): The forward propagation of the representation of the

node s from block l to block $(l+1)$ is given by

$$
\begin{aligned}
\mathbf{h}_s^{[l+1/5]} &= \boldsymbol{\gamma}_2^{[l+1]} + \boldsymbol{\Gamma}_1^{[l+1]} \mathbf{h}_s^{[l]}, \\
\mathbf{h}_s^{[l+2/5]} &= \text{BatchNorm}(\mathbf{h}_s^{[l+1/5]}), \\
\mathbf{h}_s^{[l+3/5]} &= \text{ReLU}(\mathbf{h}_s^{[l+2/5]}), \\
\mathbf{h}_s^{[l+4/5]} &= \boldsymbol{\gamma}_4^{[l+1]} + \boldsymbol{\Gamma}_4^{[l+1]} \mathbf{h}_s^{[l+3/5]}, \\
\mathbf{h}_s^{[l+1]} &= \text{ReLU}(\mathbf{h}_s^{[l+4/5]}).
\end{aligned}
$$

Note that in the Multilayer Perceptron, the graph structure is not used.

- Global pooling layer: The forward propagation of the global pooling layer is given by

$$
\begin{aligned}
\mathbf{h}^{[L+1/2]} &= (\max_s\{h_{s1}^{[L]}\}_{s=1}^S, \max\{h_{s2}^{[L]}\}_{s=1}^S, \cdots, \max\{h_{sd}^{[L]}\}_{s=1}^S), \\
\mathbf{z} &= \text{Dropout}(\mathbf{h}^{[L+1/2]}).
\end{aligned}
$$

Appendix B

Below we describe the conformal prediction approach to controlling the risk of the machine learning models. Conformal prediction aims to estimate a set $\mathcal{C}(X^{\text{test}})$ such that

$$\mathbb{P}(Y^{\text{test}} \in \mathcal{C}(X^{\text{test}})) \geq 1 - \alpha$$

holds. Below we only consider conformal prediction for the classification problem. Conformal prediction for classification is based on the following idea [9]. It constructs the prediction set $\mathcal{C}(X^{\text{test}})$ by finding a conformity score $s(X, Y)$ such that the relation

$$\mathcal{C}(X^{\text{test}}) = \{y : s(X^{\text{test}}, y) \leq q\}$$

holds. Here the value q is a threshold and can be found by investigating the distribution of the conformity scores. In practice the distribution of the conformity scores can be approximated by the empirical distribution of the conformity scores computed from the calibration set.

Conformal prediction has gained popularity in statistical inference for deep neural networks [12, 6, 10, 9, 8, 3, 2, 7, 5, 4]. [1] provided a good introduction to conformal prediction and its applications in controlling risks of various machine learning models.

In our paper we considered three methods for controlling risk of our machine learning model. The first method, Naive Prediction Set, sets the threshold value $q = 1 - \alpha$, where α is the risk control level. The other two methods, Adaptive Prediction Sets [9] and Regularized Adaptive Prediction Sets [2], are based on conformal prediction. They set the threshold value q according to the empirical distribution of the conformity scores of the calibration set. In practice, if the risk control level is α, then q is defined as the $\lceil (1 - \alpha)(n^{\text{cal}} + 1) \rceil / (n^{\text{cal}} + 1)$-quantile of the empirical distribution, where n^{cal} is the sample size of the calibration set.

Now let σ_k be the score of the kth diagnosis. Below we describe how the two methods compute the conformity scores from the calibration set:

- Adaptive Prediction Sets (APS, [9]): The corresponding conformity score is given by

$$s(x, y) = \sum_{j=1}^{k:\pi_k = y} \sigma_{\pi_j} - u \cdot \sigma_{\pi_k}.$$

where $\{\pi_j\}_{j=1}^{K}$ is a permutation of $\{1, 2, \cdots, K\}$ such that $\sigma_{\pi_1} \geq \sigma_{\pi_2} \geq \cdots \geq \sigma_{\pi_K}$, π_k is the ground truth label, and $u \sim \text{Uniform}(0, 1)$.

- Regularized Adaptive Prediction Sets (RAPS, [2]): The corresponding conformity score is given by

$$s(x, y) = \sum_{j=1}^{k:\pi_k = y} \sigma_{\pi_j} - u \cdot \sigma_{\pi_k} + \lambda(k - k_{\text{reg}})_+,$$

where $u \sim \text{Uniform}(0, 1)$, and $(\lambda, k_{\text{reg}})$ are two hyparameters that require tuning.

Appendix C

The original dataset contains 21,636 subjects of four disease types, of which 12,854 subjects were normal, 5,511 subjects were diagnosed as mild fatty liver, 2,664 were diagnosed as moderate fatty liver, and 607 were diagnosed as severe fatty liver. The original dataset were cleaned by dropping subjects using a machine learning-based procedure that aims to identify disease subjects with unknown severity. Table 1 compares the original dataset and the cleaned dataset in terms of sample sizes in the four fatty liver disease types.

Class	Uncleaned	Cleaned	Cleaned/Uncleaned
normal	12,854	12,321	95.85%
mild	5,511	2,287	41.50%
moderate	2,664	1,629	61.55%
severe	607	535	88.14%
total	21,636	16,772	77.52%

Table 1: Data sizes after data clean procedures.

Appendix D

Further results of the two-class classification under the three risk control methods:

Method	Class	Missed	Coverage	Total
NPS	Unambiguous	6.0	805.8	811.8
	Ambiguous	0	78.2	78.2
	Total	6.0	884.0	890
APS	Unambiguous	0.0	33.4	33.4
	Ambiguous	0.0	856.6	856.6
	Total	0.0	890	890
RAPS	Unambiguous	13.7	835.7	849.4
	Ambiguous	0	40.6	40.6
	Total	13.7	876.3	890

Table 2: Results of the average numbers of subjects of the unambiguous group and ambiguous group under the three risk control methods ($\alpha = 0.1$). The average numbers were calculated based on 10 replicates.

Label set	NPS		APS		RAPS	
	n_{correct}	n	n_{correct}	n	n_{correct}	n
[0]	410.5	411.7	2.1	2.1	420.4	425.4
[1]	395.3	400.1	31.3	31.3	415.3	424.0
Total	805.8	811.8	33.4	33.4	835.7	849.4
$[0, 1]$	78.2	78.2	856.6	856.6	40.6	40.6
Total	78.2	78.2	856.6	856.6	40.6	40.6

Table 3: Results of the average numbers of subjects of different classes under the three risk control methods at $\alpha = 0.1$. n_{correct} = the average number of subjects whose labels are correctly covered by the prediction sets. n = the average number of subjects. The average numbers were calculated based on 10 replicates. 0 = normal; 1 = mild, or moderate, or severe.

Further results of the three-class classification under the three risk control methods:

Method	Class	Missed	Coverage	Total
NPS	Unambiguous	11.3	356.1	367.4
	Ambiguous	3.3	277.3	280.6
	Total	14.6	633.4	648
APS	Unambiguous	0	35.5	35.5
	Ambiguous	0	612.5	612.5
	Total	0	648	648
RAPS	Unambiguous	32	433	465
	Ambiguous	3.4	179.6	183
	Total	35.4	612.6	648

Table 4: Results of the average numbers of subjects of the unambiguous group and ambiguous group under the three risk control methods ($\alpha = 0.1$). The average numbers were calculated based on 10 replicates.

Label set	NPS		APS		RAPS	
	n_{correct}	n	n_{correct}	n	n_{correct}	n
[0]	188.8	189.2	35.5	35.5	195.5	197.7
[1]	62.3	67.2	0	0	101.6	116.6
[2]	105.0	111.0	0	0	135.9	150.7
Total	356.1	367.4	35.5	35.5	433	465
$[0, 1]$	45.5	47.3	82.3	82.3	32.9	34.7
$[0, 2]$	0.7	0.8	0	0	1.3	1.6
$[1, 2]$	213.6	215.0	156.2	156.2	138.3	139.6
$[0, 1, 2]$	17.5	17.5	374.0	374.0	7.1	7.1
Total	277.3	280.6	612.5	612.5	179.6	183

Table 5: Results of the average numbers of subjects of different classes under the three risk control methods at $\alpha = 0.1$. n_{correct} = the average number of subjects whose labels are correctly covered by the prediction sets. n = the average number of subjects. The average numbers were calculated based on 10 replicates. 0 = normal; 1 = mild; 2 = moderate or severe.

Further results of the four-class classification under the three risk control methods:

Method	Class	Missed	Coverage	Total
NPS	Unambiguous	2.4	76	78.4
	Ambiguous	3.4	134.2	137.6
	Total	5.8	210.2	216
APS	Unambiguous	0.0	15.8	15.8
	Ambiguous	0.2	200	200.2
	Total	0.2	215.8	216
RAPS	Unambiguous	10.2	104.8	115
	Ambiguous	4.2	96.8	101
	Total	14.4	201.6	216

Table 6: Results of the average numbers of subjects of the unambiguous group and ambiguous group under the three risk control methods ($\alpha = 0.1$). The average numbers were calculated based on 10 replicates.

Label set	NPS		APS		RAPS	
	n_{correct}	n	n_{correct}	n	n_{correct}	n
[0]	44.4	44.4	15.8	15.8	47.2	47.5
[1]	10.4	11.1	0.0	0.0	22.1	25.9
[2]	0.0	0.1	0.0	0.0	5.0	7.1
[3]	21.2	22.8	0.0	0.0	30.5	34.5
Total	76	78.4	15.8	15.8	104.8	115
$[0, 1]$	14.0	14.5	17.1	17.1	10.2	10.8
$[0, 2]$	0.0	0.0	0.0	0.0	0.2	0.3
$[0, 3]$	0.0	0.0	0.0	0.0	0.0	0.1
$[1, 2]$	48.7	49.4	2.3	2.3	40.5	41.6
$[1, 3]$	0.0	0.0	0.0	0.0	0.0	0.0
$[2, 3]$	47.2	49.3	11.6	11.6	37.1	39.2
$[0, 1, 2]$	8.7	8.8	25.3	25.5	3.5	3.6
$[0, 1, 3]$	0.0	0.0	0.0	0.0	0.0	0.0
$[0, 2, 3]$	0.0	0.0	0.0	0.0	0.4	0.5
$[1, 2, 3]$	14.7	14.7	73.5	73.5	4.5	4.5
$[0, 1, 2, 3]$	0.9	0.9	70.2	70.2	0.4	0.4
Total	134.2	137.6	200	200.2	96.8	101

Table 7: Results of the average numbers of subjects of different classes under the three risk control methods at $\alpha = 0.1$. n_{correct} = the average number of subjects whose labels are correctly covered by the prediction sets. n = the average number of subjects. The average numbers were calculated based on 10 replicates. 0 = normal; 1 = mild; 2 = moderate; 3 = severe.

References

[1] A. N. Angelopoulos and S. Bates. A gentle introduction to conformal rrediction and distribution-free uncertainty quantification. *ArXiv*, page arXiv:2107.07511v3, 2022.

[2] A. N. Angelopoulos, S. Bates, J. Malik, and M. I. Jordan. Uncertainty sets for image classifiers using conformal prediction. *ICLR*, 2021.

[3] R. F. Barber, E. J. Candés, A. Ramdas, and Tibshirani R. J. Predictive inference with the Jackknife+. *The Annals of Statistics*, 49:486–507, 2021.

[4] E. Candés, L. Lei, and Z. Ren. Conformalized survival analysis. *ArXiv*, page arXiv:2103.09763v2, 2022.

[5] Y. Lee and R. F. Barber. Distribution-free inference for regression: discrete, continuous, and in between. *NeurIPS*, 2021.

[6] J. Lei, M. G'Sell, A. Rinaldo, R. J. Tibshirani, and L. Wasserman. Distribution-free predictive inference for regression. *Journal of the American Statistical Association*, 113:1094–1111, 2018.

[7] L. Lei and E. J. Candés. Conformal inference of counterfactuals and individual treatment effects. *Journal of the Royal Statistical Society*, 83:911–938, 2021.

[8] Y. Romano, E. Patterson, and E. J. Candés. Conformalized quantile regression. *NeurIPS*, 2019.

[9] Y. Romano, M. Sesia, and E. J. Candés. Classification with valid and adaptive coverage. *NeurIPS*, 2020.

[10] R. J. Tibshirani, R. F. Barber, E. J. Candés, and A. Ramdas. Conformal prediction under covariate shift. *NeurIPS*, 2019.

[11] P. Veličković, G. Cucurull, A. Casanova, A. Romero, P. Lió, and Y. Bengio. Graph attention networks. *ICLR*, 2018.

[12] V. Vovk, A. Gammerman, and G. Shafer. *Algorithmic Learning in a Random World.* Springer, New York, 2005.

[13] K. Xu, W. Hu, J. Leskovec, and S. Jegelka. How powerful are graph neural networks? *ICLR*, 2019.
